# Health Tracts

**Published:** 1866-04

**Authors:** 


### "HEALTH TRACTS"

Are thus noticed in one of our exchanges : "In HALL'S JOURNAL OF HEALTH, one of the best periodicals published in the United States, there can be found a series of the most valuable essays ever placed in type : they are not mere ephemeral fantasies, but practical truths, which, if read and followed carefully, will benefit the whole human race." These Health Tracts, 236 in number, with a steel engraving of the Editor, are sent, post-paid, for $2.50, by addressing "Hall's Journal of Health, No. 2 West 43d St., New-York."

Of the Book on "Health and Disease," (price $1.60 by mail) which has now passed through several editions, the manuscript of which was rejected by all the prominent publishers of New York City, a member of Congress, who has made his mark for time, says, in a letter recently received by the Publisher, "Send me another copy of *Health and Disease.* I have been so much pleased with it, that I desire to extend its usefulness."

The "Health Tracts" were offered to one of the very largest Publishing Houses in New-York, but were "very respectfully declined." It would seem from this, that there is a difference of opinion as to their value. As they have been copied, all of them, in hundreds, perhaps thousands of papers, all over the country, it may reasonably be presumed that they are of some practical value. The original intention was to print a tract on moral health, on the other side of the page of one on physical health, and thus make one preserve the other, and do good by stealth ; the Editor is thankful to possess the evidence, in letters received from all parts of the country, from persons of all classes and professions, including strangers and friends, that the mark aimed at has not been wholly missed. Each tract is eminently practical, and is contained on one page ; it is believed that they will be as useful and true in the next generation as now ; and that the increasing intelligence of the people will proportionably increase the general favorable estimate of their value.

While writing about our own books, we add something in reference to that on

CONSUMPTION, the third edition revised, with additions ($1.60 by mail) ; its object is to point out the very earliest and infallible symptoms of its first approach ; the indications of its more advanced stages; and then to show the comparatively easily practicable cure of the malady at this point of the disease, by entirely physical means, which are stated and illustrated by cases coming under the care of another physician, an Army Surgeon, in connection with corroboration under the author's care; by means of the suggestions made persons have recovered their health and lived many years afterwards; perhaps the same thing may occur again in reference to others and for this reason, it is brought distinctly into notice again especially as we believe that it is the only book yet published for popular reading which gives a truthful view of the dreadful disease.

